# Plasmids of psychrophilic and psychrotolerant bacteria and their role in adaptation to cold environments

**DOI:** 10.3389/fmicb.2014.00596

**Published:** 2014-11-06

**Authors:** Lukasz Dziewit, Dariusz Bartosik

**Affiliations:** Department of Bacterial Genetics, Faculty of Biology, Institute of Microbiology, University of WarsawWarsaw, Poland

**Keywords:** plasmid, cold environment, psychrophilic and psychrotolerant bacteria, cold adaptation, horizontal gene transfer

## Abstract

Extremely cold environments are a challenge for all organisms. They are mostly inhabited by psychrophilic and psychrotolerant bacteria, which employ various strategies to cope with the cold. Such harsh environments are often highly vulnerable to the influence of external factors and may undergo frequent dynamic changes. The rapid adjustment of bacteria to changing environmental conditions is crucial for their survival. Such “short-term” evolution is often enabled by plasmids—extrachromosomal replicons that represent major players in horizontal gene transfer. The genomic sequences of thousands of microorganisms, including those of many cold-active bacteria have been obtained over the last decade, but the collected data have yet to be thoroughly analyzed. This report describes the results of a meta-analysis of the NCBI sequence databases to identify and characterize plasmids of psychrophilic and psychrotolerant bacteria. We have performed in-depth analyses of 66 plasmids, almost half of which are cryptic replicons not exceeding 10 kb in size. Our analyses of the larger plasmids revealed the presence of numerous genes, which may increase the phenotypic flexibility of their host strains. These genes encode enzymes possibly involved in (i) protection against cold and ultraviolet radiation, (ii) scavenging of reactive oxygen species, (iii) metabolism of amino acids, carbohydrates, nucleotides and lipids, (iv) energy production and conversion, (v) utilization of toxic organic compounds (e.g., naphthalene), and (vi) resistance to heavy metals, metalloids and antibiotics. Some of the plasmids also contain type II restriction-modification systems, which are involved in both plasmid stabilization and protection against foreign DNA. Moreover, approx. 50% of the analyzed plasmids carry genetic modules responsible for conjugal transfer or mobilization for transfer, which may facilitate the spread of these replicons among various bacteria, including across species boundaries.

## Introduction

Cold environments represent a large fraction of the surface area of the Earth. They include polar regions as well as most of the oceans, which cover approximately 70% of the Earth's surface (Casanueva et al., 2010; Margesin and Miteva, 2011). Such habitats experience extreme conditions that are challenging to most life forms. However, even such harsh ecological niches are inhabited by various microorganisms, most of which are cold-adapted bacteria.

Cold-adapted bacteria can be classified into two groups based on their temperature tolerance: (i) psychrophiles, which can grow at temperatures not exceeding approx. 20°C, and (ii) psychrotrophs (or psychrotolerants), that tolerate a broader range of temperatures—between 0 and approx. 30°C (Morita, 1975). Both groups of microorganisms share basic molecular and physiological characteristics, which permit their survival in extremely cold environments: (i) increased fluidity of cellular membranes, (ii) the ability to accumulate compatible solutes (e.g., glycine, betaine and trehalose), (iii,iv) the expression of cold shock, antifreeze and ice-nucleating proteins, as well as (v) the production of cold-active enzymes (Casanueva et al., 2010).

The diversity, biology and ecology of psychrophilic or psychrotolerant bacteria have been extensively studied in recent years. Furthermore, numerous strains have been subjected to genomic investigations. This research has been mainly focused on their mechanisms of cold adaptation, which are interesting from both an evolutionary and a biotechnological point of view (Casanueva et al., 2010; Martinez-Rosales et al., 2012). Unfortunately, very little attention has been paid to the range and direction of horizontal gene transfer (HGT) among cold-active bacteria.

Extremely cold habitats are usually endemic and highly vulnerable to the influence of external factors, and therefore may undergo many dynamic changes. In such circumstances, rapid adaptation of bacterial cells to the changing environmental conditions is necessary for survival. Such “short-term” evolution is possible due to the presence of various mobile genetic elements (MGEs), including plasmids, which are considered major contributors to HGT.

Plasmids increase the plasticity of bacterial genomes, and consequently promote genetic diversity of their hosts (Siefert, 2009). Besides a set of basic structural genetic modules responsible for plasmid replication, maintenance and spread, these replicons very often carry an additional load of genetic information. These accessory genes serve as a reservoir of diverse genetic determinants whose presence may confer an evolutionary advantage to the host strains under appropriate environmental conditions (e.g., Wiedenbeck and Cohan, 2011; Heuer and Smalla, 2012).

Many plasmid-harboring bacterial strains have been isolated from permanently cold environments including the Arctic (e.g., Dziewit et al., 2013a; Moller et al., 2014), Antarctica (e.g., Kobori et al., 1984; Dziewit et al., 2013b) and Siberia (e.g., Petrova et al., 2014). Several plasmids were also identified during genomic projects of psychrophilic bacteria, e.g., five plasmids were recognized in the genome of *Carnobacterium gilichinskyi* WN1359T, a strain originating from Siberian permafrost (Leonard et al., 2013). Although plasmids appear to be common components of the genomes of psychrophilic and psychrotolerant bacteria, current knowledge about them is very fragmentary. A relatively small number have been fully sequenced and only a few analyzed in detail.

In this study we have performed a meta-analysis of the NCBI sequence databases, aimed at identifying plasmids of cold-active bacteria. The identified plasmids were thoroughly analyzed and their predicted adaptive role investigated.

## Material and methods

### Plasmid identification

The search for plasmids of cold-active bacteria was performed primarily in the NCBI databases (http://www.ncbi.nlm.nih.gov/), as well as the EMBL-EBI (http://www.ebi.ac.uk/) and Aclame databases (Leplae et al., 2010). The NCBI databases were searched using a set of words and phrases related to cold environments and cold adaptation. The genome browser in the Microbial Genomes Homepage (NCBI; http://www.ncbi.nlm.nih.gov/genome/browse/) was used to check for the presence of plasmids within the sequenced genomes of bacteria belonging to genera usually considered as psychrophilic (e.g., *Psychrobacter*, *Pseudoalteromonas*, *Psychromonas* etc). For further analyses only the plasmids of strains defined as psychrophilic or psychrotolerant according to the NCBI record or original research article were used.

### Bioinformatic analyses

Plasmid nucleotide sequences were analyzed using Artemis software (Carver et al., 2008). Similarity searches were performed using the BLAST (Altschul et al., 1997) and Pfam 27.0 programs (Finn et al., 2014). The EC numbers for particular proteins were assigned using the PRIAM tool (Claudel-Renard et al., 2003). For the functional annotation of predicted proteins and their classification into appropriate Clusters of Orthologous Groups (COGs), the Batch Web CD-Search Tool (*E* value < 1e-05) (Marchler-Bauer et al., 2011) was used. The screening of metabolic pathways was performed using the KAAS—KEGG Automatic Annotation Server (Moriya et al., 2007) and then the KEGG ORTHOLOGY (KO) Database, linking genomes to pathways by ortholog annotation (Kanehisa and Goto, 2000; Kanehisa et al., 2014). The reference data set for the computational prediction of metabolic pathways was obtained from the MetaCyc database (Caspi et al., 2014). All predictions were verified manually.

The classification of the relaxases was performed using BALSTp analysis comparing the amino acid sequence of each relaxase with the manually curated databases of the relaxases representing particular families (Garcillan-Barcia et al., 2009). Additionally within the sequence of each analyzed relaxase the conserved amino acid motifs (specific for the particular relaxase family) were identified, confirming the classification.

The analysis of predicted restriction-modification systems was performed using REBASE (The Restriction Enzyme Database) (Roberts et al., 2010). Spacer sequences of CRISPR-Cas systems were identified using the CRISPRfinder program (Grissa et al., 2007).

## Results and discussion

### Diversity and general features of plasmids of cold-active bacteria

We performed in depth searches of available DNA databases in order to identify the sequences of plasmids of psychrophilic and psychrotolerant bacteria. This analysis revealed the presence of 66 such replicons (named pPSYCH plasmids) occurring in 39 bacterial strains (Table 1) isolated from various geographical locations: Antarctica (15 plasmids), Arctic (14), France (4), Japan (2), Norway (6), Poland (2), Puerto Rico (2), Russia (9), and the USA (5). For a few strains, information concerning the site of isolation was not available. It is important to note that the nucleotide sequences of 37 plasmids were obtained as a result of whole genome sequencing projects and have not previously been analyzed in detail.

**Table 1:** **Completely sequenced plasmids of psychrophilic and psychrotolerant bacteria (pPSYCH) analyzed in this study**.

**Plasmid name**	**Host strain**	**Strain origin**	**Size (kb)**	**GC (%)**	**No. of genes**	**References/Accession No**.
pVSAL43	*Aliivibrio salmonicida* LFI1238	Norway, Atlantic cod	4.3	35.6	3	(Hjerde et al., 2008)/FM178384
pVSAL54	*A. salmonicida* LFI1238	Norway, Atlantic cod	5.4	38.1	3	(Hjerde et al., 2008)/FM178383
pVSAL320	*A. salmonicida* LFI1238	Norway, Atlantic cod	30.8	37.3	32	(Hjerde et al., 2008)/FM178382
pVSAL840	*A. salmonicida* LFI1238	Norway, Atlantic cod	83.5	40.1	72	(Hjerde et al., 2008)/FM178381
pVSAL111	*A. salmonicida* TEO83.001	Norway, Salmon salar	11.1	37.4	8	–/HG983279
pVSAL68	*A. salmonicida* Vs289	Norway, Salmon salar	6.8	38.5	5	–/HG983280
pGIAK1	*Bacillaceae* bacterium JMAK1	Antarctica, stairway of Concordia station	38.4	37.5	49	(Guo and Mahillon, 2013)/JX406743
pBWB401	*Bacillus weihenstephanensis* KBAB4	France, forest soil near Versailles	417.1	33.7	332	–/CP000904
pBWB402	*B. weihenstephanensis* KBAB4	France, forest soil near Versailles	75.1	33.4	75	–/CP000905
pBWB403	*B. weihenstephanensis* KBAB4	France, forest soil near Versailles	65.0	43.4	76	–/CP000906
pBWB404	*B. weihenstephanensis* KBAB4	France, forest soil near Versailles	52.8	35.4	71	–/CP000907
pWNCR9	*Carnobacterium gilichinskyi* WN1359T	Siberia, permafrost	9.6	29.7	14	(Leonard et al., 2013)/CP006817
pWNCR12	*C. gilichinskyi* WN1359T	Siberia, permafrost	12.7	37.0	18	(Leonard et al., 2013)/CP006813
pWNCR15	*C. gilichinskyi* WN1359T	Siberia, permafrost	15.5	34.6	18	(Leonard et al., 2013)/CP006814
pWNCR47	*C. gilichinskyi* WN1359T	Siberia, permafrost	47.1	32.3	46	(Leonard et al., 2013)/CP006815
pWNCR64	*C. gilichinskyi* WN1359T	Siberia, permafrost	64.5	34.6	62	(Leonard et al., 2013)/CP006816
plasmid small	*Desulfotalea psychrophila* LSv54	Arctic, sediments	14.7	28.6	17	(Rabus et al., 2004)/CR522872
plasmid large	*D. psychrophila* LSv54	Arctic, sediments	121.6	43.6	101	(Rabus et al., 2004)/CR522871
pEXIG01	*Exiguobacterium sibiricum* 255-15	Siberia, permafrost	4.9	37.1	5	(Rodrigues et al., 2006)/CP001023
pEXIG02	*E. sibiricum* 255-15	Siberia, permafrost	1.8	41.4	3	(Rodrigues et al., 2006)/CP001024
pCP1	*Flavobacterium psychrophilum* D12	salmonid fish	3.4	27.4	4	(Alvarez et al., 2004)/AY277637
pFL1	*Flavobacterium* sp. KP1	unknown	2.3	32.7	2	(Ashiuchi et al., 1999)/AB007196
pGLAAG01	*Glaciecola* sp. 4H-3-7+YE-5	Japan, sediments at Suruga Bay	341.3	42.0	332	(Klippel et al., 2011)/CP002527
pTA144 Dw	*Moraxella* sp. TA144	Antarctica	1.3	46.2	0	(Tutino et al., 2000)/AJ224744
pTA144 Up	*Moraxella* sp. TA144	Antarctica	1.9	40.6	1	(Tutino et al., 2000)/AJ224743
pOA307_63	*Octadecabacter antarcticus* 307	Antarctica, McMurdo Sound	62.9	52.8	60	(Vollmers et al., 2013)/CP003741
pOA238_160	*Octadecabacter arcticus* 238	USA, 150 km offshore, Alaska	159.7	54.4	157	(Vollmers et al., 2013)/CP003744
pOA238_118	*O. arcticus* 238	USA, 150 km offshore, Alaska	118.3	51.5	126	(Vollmers et al., 2013)/CP003743
pMWHK1	*Pedobacter cryoconitis* BG5	Antarctica	6.2	34.8	8	(Wong et al., 2013)/FJ613505
pPBPR1	*Photobacterium profundum* SS9	deap sea	80.0	44.0	67	(Vezzi et al., 2005)/CR377818
pMtBL	*Pseudoalteromonas haloplanktis* TAC 125	Antarctica	4.1	39.3	2^*^	(Tutino et al., 2001)/AJ224742
pKW1	*Pseudoalteromonas* sp. 643A	Antarctica, krill *Euphasia superba*	4.6	43.2	7	(Cieslinski et al., 2008)/EU636993
pSM327	*Pseudoalteromonas* sp. BSi20327	Arctic, sea ice	6.1	37.5	4	–/GU198194
pSM429	*Pseudoalteromonas* sp. Bsi429	Arctic, sea ice	3.9	28.4	4	(Zhao et al., 2011)/EU627679
pPS1M3	*Pseudoalteromonas* sp. PS1M3	deap see	3.1	37.1	1	(Kurusu et al., 2001)/AB022096
pGLE121P1	*Pseudomonas* sp. GLE121	Antarctica, glacier ice	6.9	49.7	9	(Dziewit et al., 2013b)/KC542381
pGLE121P2	*Pseudomonas* sp. GLE121	Antarctica, glacier ice	8.3	53.7	11	(Dziewit et al., 2013b)/KC542382
pGLE121P3	*Pseudomonas* sp. GLE121	Antarctica, glacier ice	39.6	52.2	44	(Dziewit et al., 2013b)/KC542383
plasmid KOPRI126573	*Pseudomonas* sp. MC1	Antarctica, waste water treatment plant	81.8	55.8	83	–/JN248563
plasmid 1	*Psychrobacter cryohalolentis* K5	Siberia, permafrost	41.2	38.3	44	–/CP000324
pKLH80	*Psychrobacter maritimus* MR29-12	Siberia, permafrost	14.8	40.3	19	(Petrova et al., 2014)/HF953351
pP109bwP1	*Psychrobacter* sp. DAB_AL109bw	Arctic, little auks guano	4.4	42.9	6	(Dziewit et al., 2013a)/JQ245702
pP12P1	*Psychrobacter* sp. DAB_AL12	Arctic, little auks guano	2.9	35.7	2	(Dziewit et al., 2013a)/JQ231228
pP32BP1	*Psychrobacter* sp. DAB_AL32B	Arctic, little auks guano	4.6	42.7	6	(Dziewit et al., 2013a)/JQ245699
pP43BP1	*Psychrobacter* sp. DAB_AL43B	Arctic, little auks guano	4.4	37.2	6	(Dziewit et al., 2013a)/JQ245700
pP43BP2	*Psychrobacter* sp. DAB_AL43B	Arctic, little auks guano	5.4	37.3	6	(Dziewit et al., 2013a)/JQ245701
pP43BP3	*Psychrobacter* sp. DAB_AL43B	Arctic, little auks guano	5.0	39.2	7	(Dziewit et al., 2013a)/JQ348845
pP43BP4	*Psychrobacter* sp. DAB_AL43B	Arctic, little auks guano	6.5	41.3	9	(Dziewit et al., 2013a)/JQ348844
pP60P1	*Psychrobacter* sp. DAB_AL60	Arctic, little auks guano	5.4	41.7	8	(Dziewit et al., 2013a)/JQ245703
pP60P2	*Psychrobacter* sp. DAB_AL60	Arctic, little auks guano	14.9	37.8	13	(Dziewit et al., 2013a)/JQ245704
pP62BP1	*Psychrobacter* sp. DAB_AL62B	Arctic, little auks guano	34.5	36.5	33	(Lasek et al., 2012)/JQ065022
plasmid PsyG_3	*Psychrobacter* sp. G	Antarctica, King George Island	4.0	38.5	5	(Che et al., 2013)/CP006268
plasmid PsyG_4	*Psychrobacter* sp. G	Antarctica, King George Island	4.5	36.5	5	(Che et al., 2013)/CP006267
plasmid PsyG_26	*Psychrobacter* sp. G	Antarctica, King George Island	26.1	41.2	26	(Che et al., 2013)/CP006266
pRWF101	*Psychrobacter* sp. PRwf-1	Puerto Rico, fish *Lutjanus vivanus*	14	38.3	15	–/CP000714
pRWF102	*Psychrobacter* sp. PRwf-1	Puerto Rico, fish *Lutjanus vivanus*	2.1	40.4	1	–/CP000715
pRUNSL01	*Runella slithyformis* DSM 19594	USA, University Lake near Baton Rouge	107.0	46.3	88	(Copeland et al., 2012)/CP002860
pRUNSL02	*R. slithyformis* DSM 19594	USA, University Lake near Baton Rouge	93.5	41.4	91	(Copeland et al., 2012)/CP002861
pRUNSL03	*R. slithyformis* DSM 19594	USA, University Lake near Baton Rouge	66.9	40.8	70	(Copeland et al., 2012)/CP002862
pRUNSL04	*R. slithyformis* DSM 19594	USA, University Lake near Baton Rouge	44.8	43.2	46	(Copeland et al., 2012)/CP002863
pRUNSL05	*R. slithyformis* DSM 19594	USA, University Lake near Baton Rouge	38.8	44.2	32	(Copeland et al., 2012)/CP002864
pSFKW33	*Shewanella* sp. 33B	Poland, Baltic Sea	8.0	38.3	8	(Werbowy et al., 2009)/FJ626843
pSinA	*Sinorhizobium* sp. M14	Poland, Zloty Stok gold mine	108.9	59.5	102	(Drewniak et al., 2013)/JF809815
plasmid F	*Sphingopyxis alaskensis* RB2256	USA, sea water near Alaska	28.5	60.4	30	(Lauro et al., 2009)/CP000357
pSP01	*Streptomyces* sp. PAMC26508	Antarctica, lichen *Cladonia borealis*	104.0	68.5	104	–/CP003991
pSCD	*Sulfuricella denitrificans* skB26	Japan, freshwater lake	86.6	56.7	91	(Watanabe et al., 2014)/AP013067

*The data were obtained from the GenBank databases (NCBI)*.

^*^all ORFs were distinguished in the course of this study (plasmid sequence deposited at the NCBI database has not been annotated).

**Figure 1:**
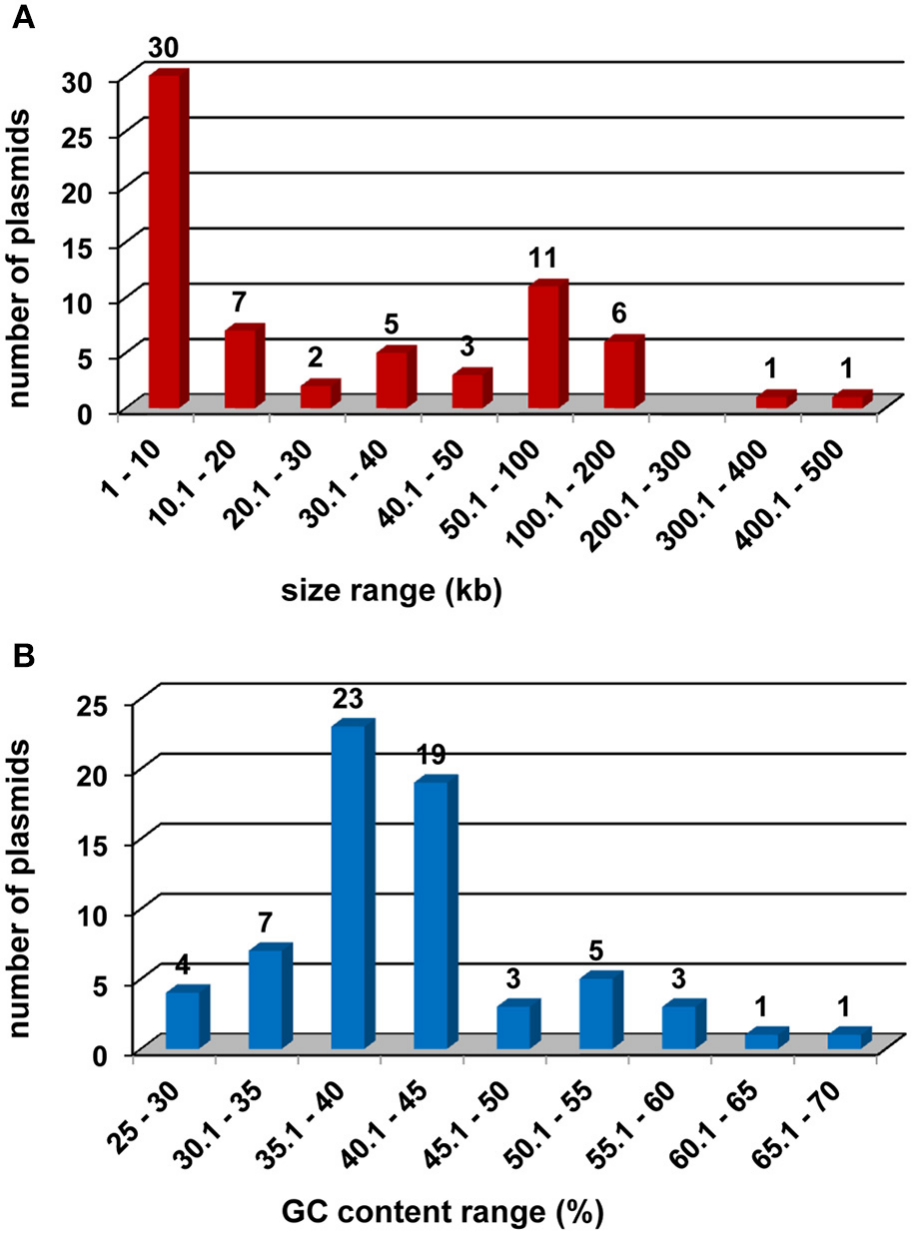
**Size range (A) and GC content range (B) of plasmids of cold-active bacteria**.

The majority of the identified plasmids (53; 80.3%) occur in Gram-negative bacteria and the most numerous group of the replicons (17; 25.8%) was found in strains of the genus *Psychrobacter* (*Gammaproteobacteria*) (Table 1). The plasmids range in size from 1.3 kb (pTA144 Dw of *Moraxella* sp. TA144) to 417 kb (pBWB401 of *Bacillus weihenstephanensis* KBAB4) (Table 1), and they contain between 0 (pTA144 Dw of *Moraxella* sp. TA144) and 332 (pGLAAG01 of *Glaciecola* sp. 4H-3-7+YE-5) open reading frames (ORFs). Many of the plasmids (30; 45.5%) are small, cryptic replicons not exceeding 10 kb in size (Figure 1A). The average GC content of the pPSYCH nucleotide sequences ranges from 27.4 to 68.5%. This GC value is <45% in 53 (80.3%) plasmids, and >55% in only 5 (7.6%) replicons (Figure 1B).

**Figure 2:**
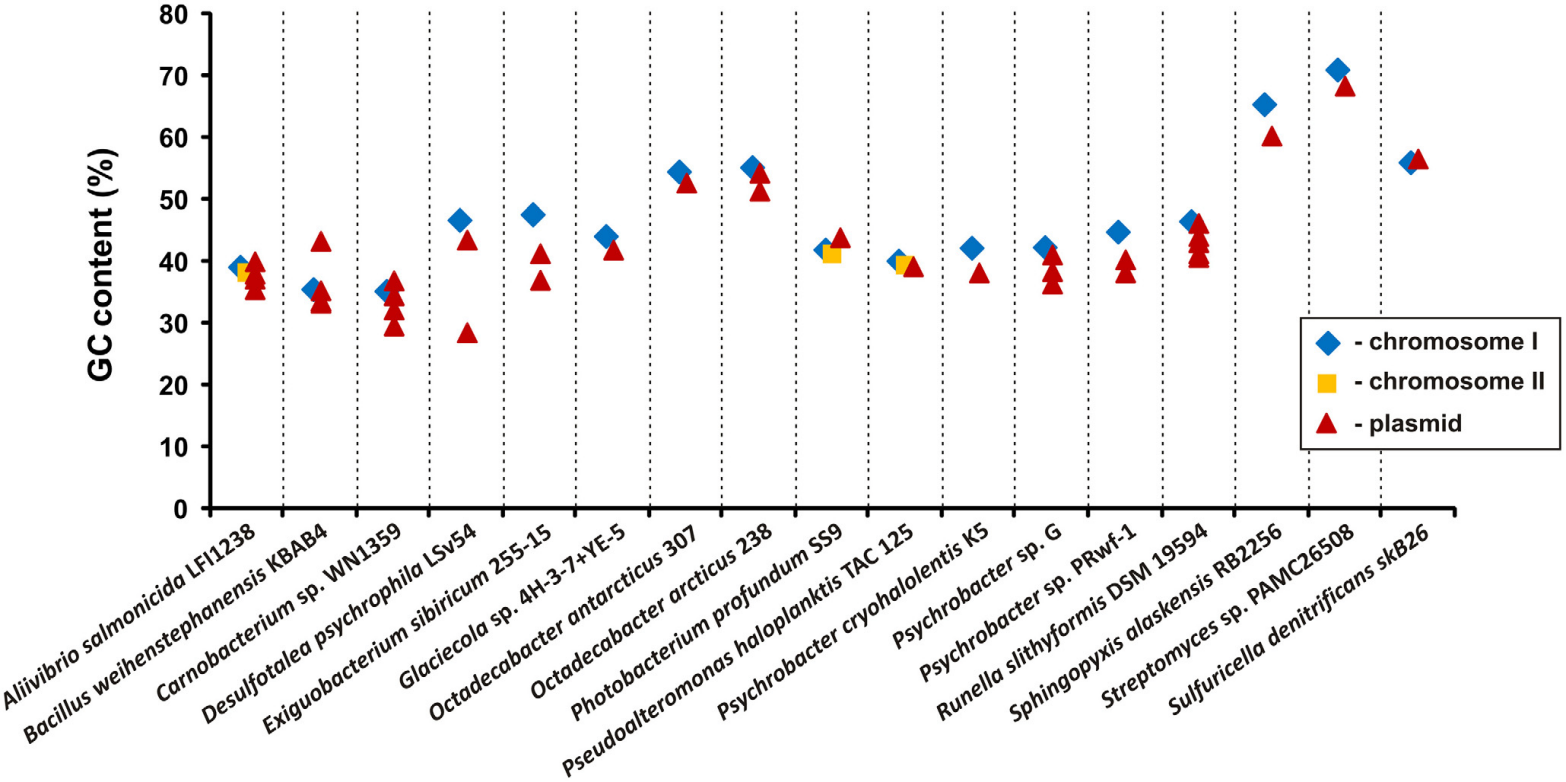
**Comparison of the GC content of the nucleotide sequences of plasmids of cold-active bacteria and their hosts chromosomes**.

A more complex analysis was performed to compare the relative GC contents of the plasmids and the chromosomes of their hosts. For this analysis we used the completely sequenced genomic DNA of 17 bacterial strains, containing 37 pPSYCH plasmids. The analysis revealed that 32 (86.5%) of the plasmids have a lower GC content than their host's chromosome. The greatest difference (18.2%) was observed between “plasmid small” and the chromosomal DNA of *Desulfotalea psychrophila* LSv54 (Figure 2). This finding is in good agreement with the observation that bacteria usually fail to stably maintain horizontally-acquired DNAs whose GC content is considerably higher than that of the chromosome (Nishida, 2012).

In the next step of the pPSYCH plasmids analysis we performed the characterization of their conserved backbones, composed of replication and conjugal transfer modules. The replication machinery is a crucial component of each plasmid, since it is responsible for its maintenance in bacterial host cells (Thomas, 2000). Therefore, for the initial classification of pPSYCH plasmids we analyzed their replication modules.

The analysis revealed that 56 plasmids carry a single replication modules, while plasmid 1 of *Psychrobacter cryohalolentis* K5 and pBWB402 of *B. weihenstephanensis* KBAB4 are composite replicons and carry two replication systems. We were not able to distinguish the replication modules of 8 pPSYCH plasmids.

We found that 29 (43.9%) plasmids encode the RepB replication protein of the Rep3 superfamily, and the structure of their replication systems is typical for many theta-replicating plasmids (Chattoraj, 2000). The replication modules of the second most numerous group of plasmids (8; 12.1%) contain a single gene encoding a putative protein with three conserved regions: the replicase domain, an alpha helical domain of primases (PriCT-1) and a HTH motif. Moreover, their predicted *oriV* regions have the structure typical for the ColE2-type plasmids (Dziewit et al., 2013a).

Analysis of the remaining plasmids revealed that they encode replication proteins which were classified into the following families: (i) RepL (2 plasmids), (ii) RepZ (ColIB-P9) (3), (iii) RepC of *repABC* system (3), (iv) replication initiation protein containing predicted helix-turn-helix (HTH_36) domain (2), (v) Rep1 (rolling circle replication initiator protein) (1), (vi) RstA (phage-related replication proteins) (1), and (vii) II/X (phage/plasmid replication protein) (1). Moreover, 10 plasmids encode unclassified replication initiation proteins.

Many plasmids are capable of horizontal transfer by conjugation. According to their transfer machineries, they may be grouped into two categories comprising self-transmissible (conjugative) and mobilizable replicons (Garcillan-Barcia et al., 2009). However, it is worth mentioning, that the conjugal transfer phenotype of a particular plasmid depends not only on the genes it carries, but also the genomic background (i.e., other co-residing replicons) (Torres Tejerizo et al., 2014).

The conjugative plasmids encode a large set of proteins responsible for the processing of conjugative DNA (MOB) and mating pair formation (MPF), while the mobilizable plasmids carry only MOB regions, composed of an origin of transfer *oriT* and a gene encoding relaxase, which nicks DNA at the *oriT* sites. The transfer of mobilizable plasmids requires a membrane-associated mating pair formation complex, which may be provided by self-transmissible plasmids or integrative and conjugative elements (ICE) (Garcillan-Barcia et al., 2009; Smillie et al., 2010). ICE primarily reside in a host chromosome but they can excise and form plasmid-like circular molecules that are intermediates for conjugal transfer. In contrast to plasmids, ICEs do not contain their own replication systems (Wozniak and Waldor, 2010).

Our analysis revealed that 32 of the pPSYCH plasmids (48.5%) carry conjugal transfer modules. Of these, 12 (18.2%) are predicted to be conjugative plasmids (size range 38.4–121.6 kb) and 20 (30.3%), mobilizable replicons (sizes not exceeding 28.5 kb).

**Figure 3:**
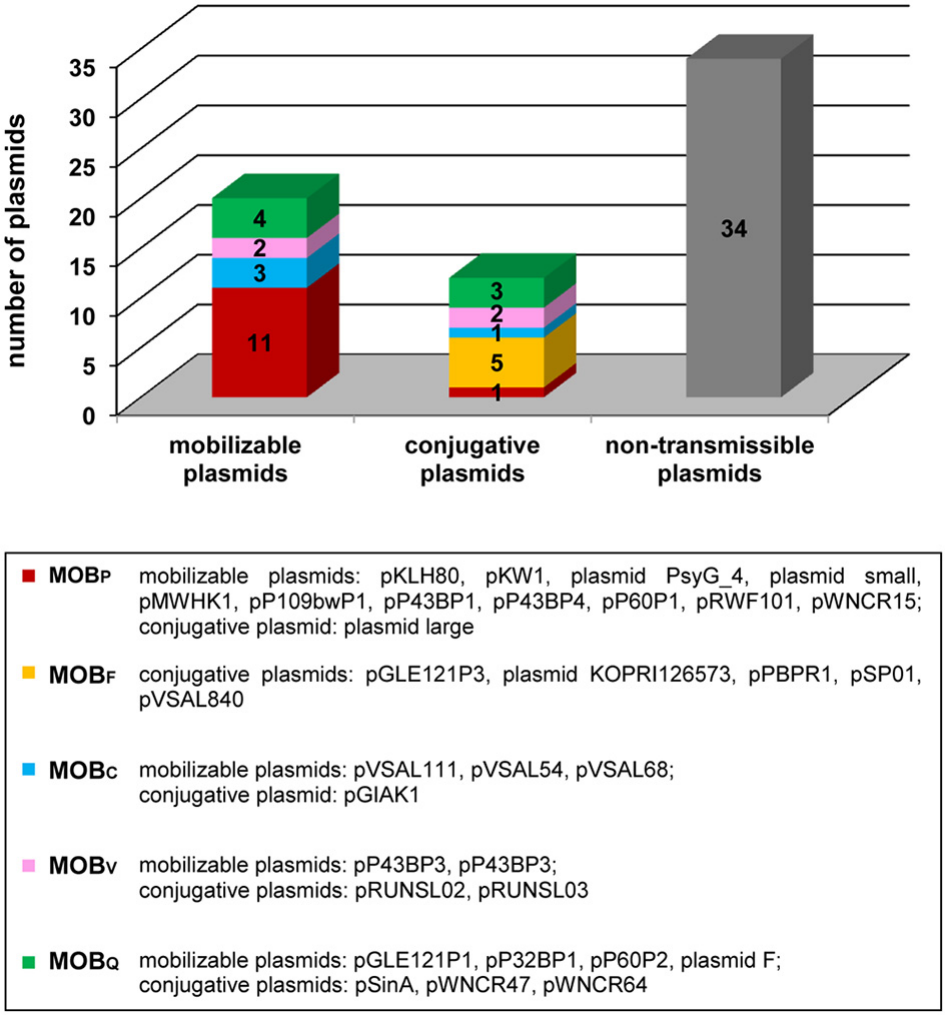
**Distribution and classification of relaxases encoded within the pPSYCH plasmids**.

Based on a detailed comparative analysis of the relaxase amino acid sequences, we found that these proteins (and the MOB modules encoding them) can be classified within five different families: MOB_P_, MOB_Q_, MOB_V_, MOB_C_ and MOB_F_. As shown in Figure 3, members of the MOB_P_family are the most numerous. It is also important to note that the relaxases of the MOB_F_ family are exclusively present in large self-transmissible plasmids. Both observations are in a good agreement with the results of a complex survey of the sequences of plasmid-encoded MOB modules (Smillie et al., 2010).

The presence of TRA and/or MOB modules within plasmid genomes (including those of cold-active bacteria) may facilitate dissemination of these replicons among evolutionarily-distinct bacterial species. Our analyses have revealed that the conjugative and mobilizable pPSYCH plasmids carry numerous genes of adaptive value, and therefore their transfer may strongly influence adaptation of the host bacteria to the harsh environmental conditions they encounter.

**Figure 4:**
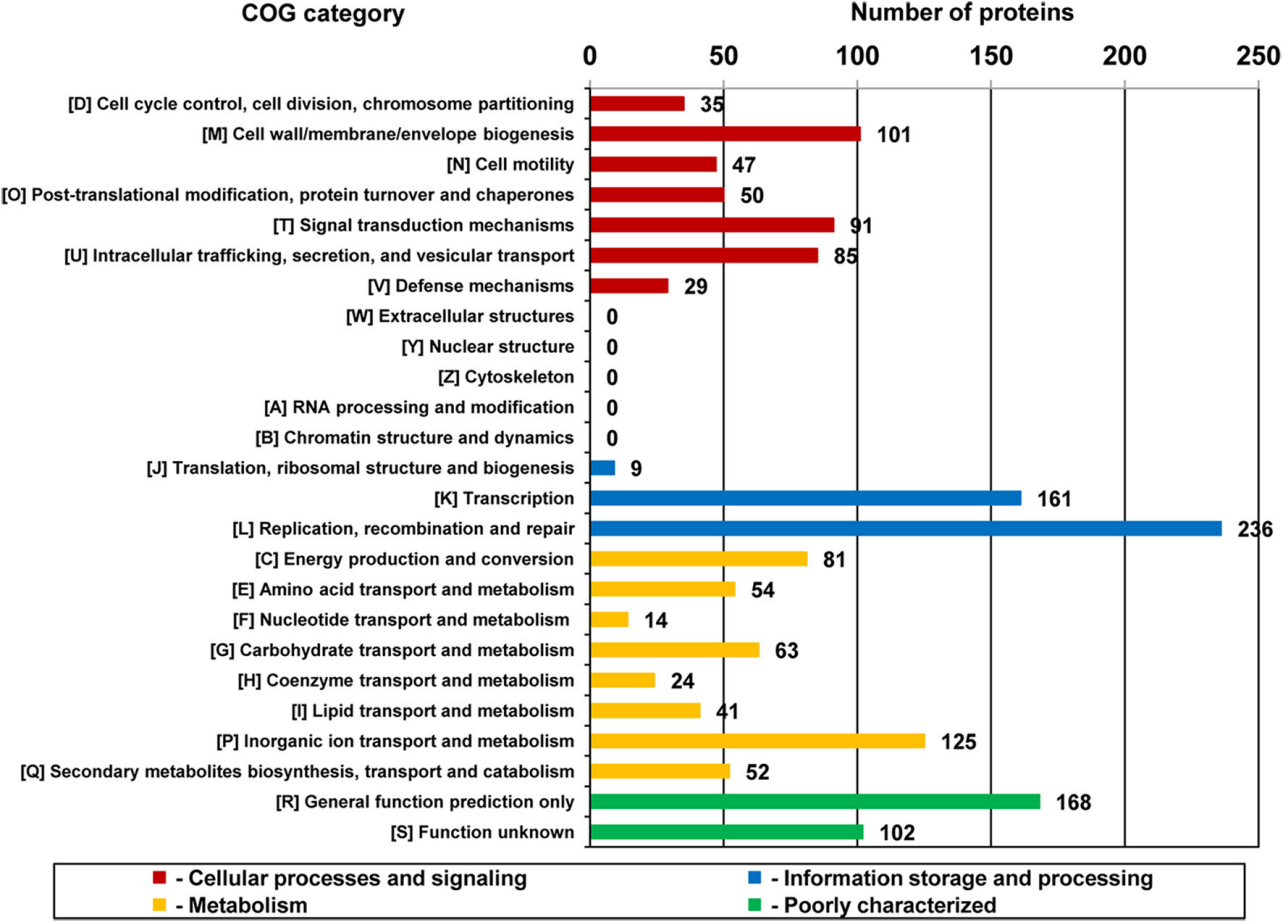
**Number of proteins encoded within the plasmids of cold-active bacteria associated with the general COG functional categories**.

Plasmids are natural genetic vectors, which strongly influence bacterial evolution by disseminating genetic information determining various phenotypes (e.g., Harrison and Brockhurst, 2012). To investigate the adaptive role of pPSYCH plasmids, functional categorization of the proteins encoded by these replicons was performed. We were able to assign COG numbers to approximately 50% of the predicted proteins and calculate the proportion of the proteins in each COG category (Figure 4, Table S1).

The largest fraction of classified proteins (15.1%) was assigned to the L category by COG annotation (Figure 4). This group gathers proteins involved in replication, recombination and repair that may be directly linked with both plasmid maintenance and the mobility of transposable elements (TEs) carried by plasmids. Our finding is in agreement with the results of previous global plasmid relationships analyses, performed with the application of gene-sharing networks, which revealed that the majority of genes shared by plasmids encode proteins related to the process of HGT itself (DNA replication and transposition) (Tamminen et al., 2012).

A significant proportion of the analyzed proteins were assigned to the K COG category, gathering proteins associated with transcription (Figure 4). Proteins of this type are often involved in the stable maintenance of bacterial plasmids (e.g., components of toxin-antitoxin systems) and overall transcriptional regulation.

A total of 454 (29%) of the pPSYCH-encoded proteins (with assigned COG numbers) appear to be involved in the overall cellular metabolism. The most numerous of these are members of COG category P, gathering proteins involved in inorganic ion transport and metabolism, while others were assigned to functional categories C, G, E, F, H, I, and Q (Figure 4).

A similar number of proteins (438; 27.9%) were assigned to categories D, M, N, O, T, U, V, whose members are involved in various cellular processes and signaling, including cell wall/membrane/envelope biogenesis (category M—the most highly represented), cellular defense (V), but also cell motility (N) and secretion (U) (Figure 4).

These data strongly suggest that plasmids of psychrophilic and psychrotolerant bacteria (as plasmid mobilome of any particular ecological niche) may determine or modulate different metabolic properties of their hosts and influence the overall functioning of bacterial cells.

### Cold protection

One of the most prominent responses of bacteria to low temperatures is the induction of cold shock protein (CSP) synthesis, which counteracts the harmful effects of the temperature downshift. The proteins comprising this diverse group include helicases, nucleases and ribosome-associated components that directly or indirectly interact with DNA and RNA. Several of these proteins are essential for survival in the cold, but none participates exclusively in cold adaptation (Weber and Marahiel, 2003).

A comparative BLASTp analysis of two manually curated databases was performed to identify cold shock proteins. One database represented a summarized proteome of all the plasmids analyzed in this study and the second included the sequences of 31 known cold shock proteins (Gualerzi et al., 2003; Barria et al., 2013). This comparative analysis revealed the presence of three types of predicted plasmid-encoded cold shock protein: (i) putative pyruvate dehydrogenases, (ii) global DNA-binding transcriptional regulators (H-NS), and (iii) nucleoid-associated proteins (HUβ).

The role of pyruvate dehydrogenase in cold adaptation is not well understood. It was proposed that this enzyme is involved in the intensification of glycolysis and suppression of the tricarboxylic acid cycle, which may lead to the retardation of cell growth, and adaptation to extreme conditions (Qiu et al., 2006). Pyruvate dehydrogenase (E1), together with two other enzymes, dihydrolipoamide acetyltransferase (E2) and dihydrolipoyl dehydrogenase (E3), forms the pyruvate dehydrogenase complex (PDC), which converts pyruvate into acetyl-CoA in a process called pyruvate decarboxylation (de Kok et al., 1998). The PDC components E1 and E2 (AceEF, DPPB38-40) were found to be encoded by “plasmid large” of *D. psychrophila* LSv54. Moreover, our analysis revealed that plasmid pOA238_160 of *Octadecabacter arcticus* 238 encodes a predicted protein, designated OA238_160p1370, with 71% amino-acid sequence identity to an E1 component-like protein of the *Hyphomonas jannaschiana* VP2 pyruvate dehydrogenase (AceE protein, EC 1.2.4.1). Interestingly, the gene encoding OA238_160p1370 (predicted AceE protein) is flanked within the pOA238_160 genome by insertion sequences, which suggests that it has been horizontally acquired.

Two other types of plasmid-encoded cold shock protein (H-NS and HUβ) most probably participate in the modulation of DNA supercoiling. It is well known that negative DNA supercoiling is increased when cells are exposed to cold shock. The reorganization of DNA structure may influence the pattern of gene expression and lead to remodeling of the metabolic state of the cell, which is crucial under stress conditions (Mizushima et al., 1997; Giangrossi et al., 2002). Detailed inspection of the plasmids' proposed proteomes revealed the presence of one H-NS family histone-like protein (OA238_118p0910 of pOA238_118) and two HUβ nucleoid-associated proteins (Glaag_4392 of pGLAAG01 and OA238_160p1030 of pOA238_160).

### Ultraviolet (UV) radiation protection

Bacteria inhabiting polar regions have to cope not only with cold, but also with increased solar UV radiation due to stratospheric ozone depletion (Madronich, 1994). UV-B exposure may cause DNA damage and increased production of reactive oxygen species (ROS), which can further induce mutagenesis and ultimately lead to genomic instability (Santos et al., 2012). We have investigated whether plasmids of cold-active bacteria have the potential to provide UV protection via mechanisms such as ROS scavenging.

Two highly homologous genetic modules are involved in conferring tolerance to UV radiation—the *rulAB* and *umuDC* DNA repair operons. Both are components of the global SOS system and therefore their expression is regulated by the transcriptional repressor LexA (Sundin and Murillo, 1999; Kim and Sundin, 2000). In response to DNA damage, proteins encoded by the operons regulate DNA synthesis and function as a DNA polymerase that facilitates replication over any DNA lesions. Therefore, both modules play an important role in maintaining of genome stability and therefore they increase the evolutionary fitness of their hosts (Tark et al., 2005). It was also shown that elevated expression of the the *umuDC* gene products confer a cold-sensitive growth phenotype that correlates with a rapid inhibition of DNA synthesis (Marsh and Walker, 1985). A crucial role seems to play UmuC, since alterations in the protein sequence resulted in loss of the cold-sensitive phenotype (Beuning et al., 2006). Further studies are necessary to understand the molecular basis and the importance of the observed phenotypes.

The aforementioned operons were identified within three of the analyzed plasmids: (i) pGLE121P3 of Arctic *Pseudomonas* sp. strain GLE121 (*rulAB*), (ii) plasmid KOPRI126573 of Antarctic *Pseudomonas* sp. strain MC1 (*pYIC1_09- pYIC1_10*) and (iii) pSCD of *Sulfuricella denitrificans* skB26 (*SCD_n03037-SCD_n03038*), originating from Japan (Table 1).

Bacteria respond to increased ROS production by the expression of alkyl hydroperoxide reductase complexes, AhpCD or AhpCF, which serve as important antioxidants. The AhpC component, considered to be the major scavenger of ROS produced by aerobic metabolism (Steele et al., 2010), is encoded by plasmid pP60P2 of Arctic *Psychrobacter* sp. strain DAB_AL60. Acquisition of the *ahpC*-encoding plasmid may have increased the adaptation abilities of the host strain to the polar environment with enhanced UV radiation (Dziewit et al., 2013a).

### Basic cellular metabolism

**Figure 5:**
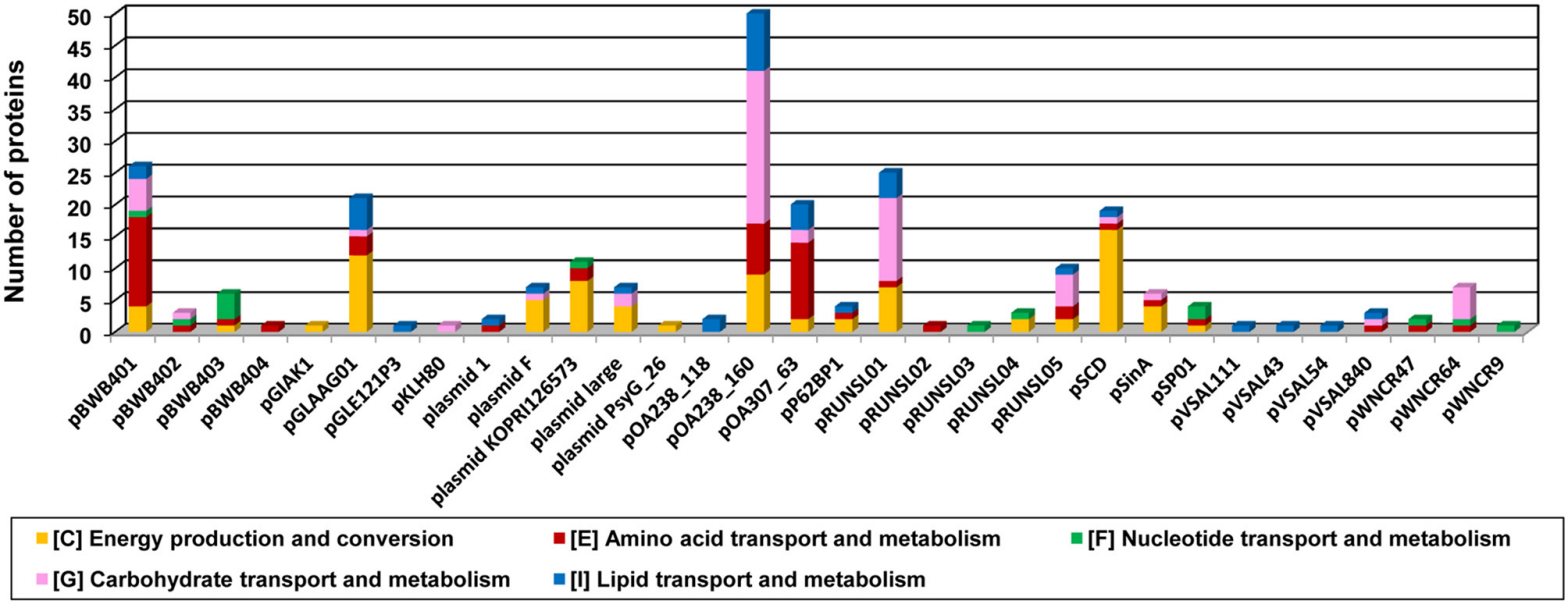
**Distribution of plasmid-encoded proteins predicted to be involved in amino acid (COG functional category E), nucleotide (F), carbohydrate (G) and lipid (I) transport and metabolism, as well as energy production and conversion (C)**.

The COG categorization of the pPSYCH-encoded proteins revealed that nearly one third of those with assigned COG numbers are predicted to be involved in cellular metabolism, including amino acid (COG functional category E), nucleotide (F), carbohydrate (G) and lipid (I) transport and metabolism, as well as energy production and conversion (C) (Figure 5). The proteins (in number from 1 to 50, depending on the replicon) classified into these COG categories were encoded within 32 plasmids (Figure 5, Table S1).

One of the pPSYCH plasmids, pOA238_160 of *O. arcticus* 238 (Table 1), is of particular interest because it encodes as many as 50 proteins, which may be directly linked with cellular metabolism (COG functional categories E, F, G, I or C). Almost 50% of these proteins appear to be involved in carbohydrate transport and metabolism. Analysis of the genetic content of this plasmid also revealed the presence of genes encoding (i) DctPQM periplasmic solute transporters of the C4-dicarboxylates malate, succinate, and fumarate (4 copies of each gene—OA238_160p0130, OA238_160p0140, OA238_160p0150; OA238_160p0940, OA238_160p0950, OA238_160p0960; OA238_160p1290, OA238_160p1300, OA238_160p1310; OA238_160p1460, OA238_160p1470, OA238_160p1480) (Forward et al., 1997); (ii) two enzymes of the citrate (TCA) cycle—aconitate hydratase (OA238_160p0470; EC 4.2.1.3), responsible for the bidirectional conversion of citrate into isocitrate, and succinate dehydrogenase (OA238_160p0460; EC 1.3.5.1), catalyzing the bidirectional transformation of succinate into fumarate; (iii) alpha-ketoglutarate-dependent taurine dioxygenase TauD (OA238_160p0180; EC 1.14.11.17), responsible for taurine utilization under sulfur starvation conditions, resulting in the release of sulfites (Eichhorn et al., 1997), and (iv) histidinol dehydrogenase HisD (OA238_160p0290; EC 1.1.1.23), catalyzing the final reaction in histidine biosynthesis (Thoma et al., 1998).

The most numerous proteins encoded by the pPSYCH plasmids are carbohydrate (COG category G) and amino-acid (COG category E) transporters and enzymes involved in the metabolism of these compounds. The G category is mainly represented by carbohydrate transporters, while the E category proteins include a broad spectrum of enzymes involved in the transport, biosynthesis and degradation of amino acids. COG functional category E representatives include, among others, four enzymes encoded within a single plasmid—pBWB401 of *Bacillus weihenstephanensis* KBAB4: (i) asparagine synthase (glutamine-hydrolysing) (BcerKBAB4_5684; EC 6.3.5.4), (ii) alanine dehydrogenase (BcerKBAB4_5593; EC 1.4.1.1), (iii) L-serine/L-threonine ammonia-lyase (BcerKBAB4_5570; EC 4.3.1.17) and (iv) homocysteine S-methyltransferase (BcerKBAB4_5466; EC 2.1.1.10) (Table S1).

These findings suggest that an efficient amino acid metabolism plays a very important role in cold-active bacteria. In nutrient-limiting cold environments, amino acids can be used as carbon and nitrogen sources, and therefore enhanced proteolytic capabilities may be beneficial to bacteria. This would explain the presence of plasmid-encoded enzymes involved in the degradation of amino acids.

Increased intracellular protein degradation has been observed in bacterial cells exposed to cold-shock (Jozefczuk et al., 2010). It seems obvious that the synthesis of induced cold shock proteins is more efficient when the availability of amino acids is increased. Therefore, the presence of additional, plasmid-encoded enzymes involved in amino acid synthesis may also be a beneficial feature.

A significant fraction of the pPSYCH-encoded proteins were identified as enzymes responsible for energy production and conversion (COG functional category C). When analyzing the distribution of genes encoding proteins of this group, we identified three plasmids carrying gene clusters encoding Ni, Fe-hydrogenases: (i) pGLAAG01 of *Glaciecola* sp. 4H-3-7+YE-5, (ii) pRUNSL01 of *Runella slithyformis* DSM 19594 and (iii) plasmid F of *Sphingopyxis alaskensis* RB2256 (Table 1). Such oxidative enzymes catalyze both the production and the consumption of hydrogen, and provide a redox mechanism by which hydrogen-metabolizing microorganisms can store and utilize energy (Fritsch et al., 2013). Since H_2_ oxidation may contribute significantly to the bacterial energy budget (Anantharaman et al., 2013), it is probable that the plasmid-encoded hydrogenases play an important role by providing an alternative and additional mechanism of energy production for bacteria inhabiting extreme environments.

### Utilization of toxic organic compounds

Increasing anthropogenic activity in cold environments results in the growing contamination of these regions with various toxic organic compounds, including long-chain and polycyclic aromatic hydrocarbons (PAH) (e.g., Aislabie et al., 1999; Macdonald et al., 2005). However, some specialized bacteria are able to utilize such compounds as carbon, nitrogen and energy sources. Therefore, the acquisition of genetic information enabling the degradation of toxic organic compounds is highly beneficial, especially under nutrient limitation, which often occurs in extremely cold environments.

Detailed analysis of the genetic content of the pPSYCH plasmids identified one replicon, KOPRI126573 of Antarctic *Pseudomonas* sp. strain MC1, containing a complete set of genes responsible for the utilization of naphthalene. This self-transferable plasmid of the IncP-9 incompatibility group is almost identical (99% nucleotide sequence identity over its entire length) to the well characterized catabolic plasmid NAH7 of *Pseudomonas putida* G7 (Dunn and Gunsalus, 1973; Sota et al., 2006). A 27-kb region of plasmid KOPRI126573 (33% of the plasmid genome) is composed of two clusters of genes, encoding (i) the upper-pathway enzymes, involved in the conversion of naphthalene to salicylate and (ii) the lower-pathway enzymes, responsible for the conversion of salicylate to acetyl-CoA *via* meta-ring cleavage (Figure S1). Similar *nah*-like gene clusters have previously been identified in several other strains of *Pseudomonas* (Habe and Omori, 2003).

### Resistance to heavy metals, metalloids and antibiotics

Environmental contamination with heavy metals and metalloids is a very common phenomenon. This is a consequence of both natural processes (e.g., occurrence of metal-containing minerals and their geobiochemical transformations, volcanic emissions, forest fires, deep-sea vents and geysers) and anthropogenic activities (e.g., large-scale burning of fossil fuels, mining, metal manufacturing, paint production and tanneries) (Adriano, 2001). Another emerging problem is the increase in antibiotics and other antimicrobial agents, originating from the medical (including veterinary) and agricultural sectors, contaminating the natural environment (Hektoen et al., 1995; Macdonald et al., 2005; Cantas et al., 2013). To combat the toxicity of these compounds bacteria develop or, more often, acquire (*via* HGT) various genetic modules conferring resistance. This phenomenon is also observed in extremely cold environments (e.g., Moller et al., 2014).

We investigated the potential of pPSYCH replicons for the determination of resistance phenotypes and identified 10 (15.2%) plasmids (Table 2) containing modules conferring heavy metal/metalloid resistance (7 replicons) or antibiotic resistance (2 replicons). One plasmid contained both types of module (Table 2). Interestingly, 8 of the plasmids carried two or more different resistance modules and may be defined as multi-resistance plasmids.

**Table 2:** **The pPSYCH plasmids containing heavy metal, metalloid and antibiotic resistance determinants**.

**Plasmid name**	**Heavy metal or metalloid resistance [locus_tag or gene name]**	**Antibiotic resistance [locus_tag or gene name]**
pGIAK1	ArsDRKRBC—arsenite and arsenate resistance [pGIAK1_24-29]	N/A
	CadDCA—cadmium resistance [pGIAK1_32-34; pGIAK1_39]	
pGLAAG01	MerRTPCA—mercury resistance [Glaag_4409-4413]	N/A
	CnrA—nickel and cobalt resistance [Glaag_4419; Glaag_4433; Glaag_4453; Glaag_4522]	
	CzcD—cobalt, zinc and cadmium resistance [Glaag_4423; Glaag_4425; Glaag_4457; Glaag_4459]	
	CopABCD—copper resistance [Glaag_4445-4448; Glaag_4478-4479; Glaag_4518-4519]	
	Copper exporting ATPase (ZntA family)—copper resistance [Glaag_4490]	
pKLH80	N/A	Carbenicillin-hydrolysing betta-lactamase-class A penicillinase—beta-lactams resistance [*blaRTG-6*]
		Streptomycin phosphotransferase—streptomycin resistance [*strAB*]
		Tetracycline efflux protein of class H—tetracycline resistance [*tetH*]
pRUNSL02	Copper exporting ATPase (ZntA family)—copper resistance [Runsl_5918; Runsl_5924]	N/A
	Copper-(or silver)-translocating P-type ATPase—copper (or silver) resistance [Runsl_5929]	
pSCD	CzcD—cobalt, zinc and cadmium resistance [SCD_n03083]	N/A
	Arsenite efflux pump (ACR3 family)—arsenite resistance [SCD_n03106]	
pSinA	ArsRCBH—arsenite and arsenate resistance [*arsRCBH*]	N/A
	CzcD—cobalt, zinc and cadmium resistance [*czcD*]	
	MerRTPA—mercury resistance [*merRTPA*]	
pSP01	N/A	Virginiamycin B hydrolase—virginiamycin resistance [F750_7148]
pWNCR12	CadD—cadmium resistance [Q783_11480]	N/A
pWNCR15	Heavy metal-(Cd/Co/Hg/Pb/Zn)-translocating P-type	Chloramphenicol acetyltransferase—chloramphenicol resistance [Q783_11585]
	ATPase—cadmium, cobalt, mercury, lead and zinc rsistance [Q783_11505]	
	CzcD—cobalt, zinc and cadmium resistance [Q783_11515]	
pWNCR64	Copper exporting ATPase (ZntA family)—copper resistance [Q783_11905]	N/A
	Copper-(or silver)-translocating P-type ATPase—copper (or silver) resistance [Q783_11920]	

Only four of the identified resistance plasmids have been thoroughly analyzed previously. The ability to confer resistance to various heavy metals or metalloids was demonstrated for pGIAK1, originating from the obligate alkaliphilic and halotolerant *Bacillaceae* strain JMAK1, pSinA of arsenite oxidizing *Sinorhizobium* sp. M14 and pSCD of sulfur oxidizer *S. denitrificans* skB26 (Drewniak et al., 2013; Guo and Mahillon, 2013; Watanabe et al., 2014); while pKLH80 of *Psychrobacter maritimus* MR29-12, a strain isolated from ancient permafrost, is a novel multidrug-resistant plasmid, carrying genes determining streptomycin, tetracycline and β-lactam resistance (Petrova et al., 2014) (Table 2).

The nucleotide sequences of the remaining pPSYCH resistance plasmids (pGLAAG01, pRUNSL02, pSP01, pWNCR12, pWNCR15, and pWNCR64) were determined in the course of several whole genome sequencing projects and these plasmids have not previously been described. We found that plasmid pGLAAGO1 of *Glaciecola* sp. 4H-3-7+YE-5 carries as many as 13 predicted heavy metal resistance modules, conferring resistance to cadmium, cobalt, copper, mercury and zinc. The only replicon that appears to determine resistance to both heavy metals and an antibiotic (chloramphenicol) is pWNCR15 of *C. gilichinskyi* WN1359T (Table 2). Interestingly, the strain WN1359T also carries two other plasmids, pWNCR12 and pWNCR64, containing heavy metal resistance modules (Table 2). The remaining two pPSYCH plasmids, pRUNSL02 (*R. slithyformis* DSM 19594) and pSP01 (*Streptomyces* sp. PAMC26508), may confer resistance to copper and virginiamycin, respectively (Table 2).

We investigated whether the identified resistance modules are associated with transposable elements and we found that the *merRTPCA* gene cluster of pGLAAG01 is most probably a component of a Tn*3*-family transposon. Moreover, it was previously shown that insertion sequence IS*Ppy1* of pKLH80 can mobilize for transposition streptomycin (*strAB*), tetracycline (*tetH*) and β-lactam (*bla*_*RTG*−6_) resistance modules (Petrova et al., 2009, 2014). The presence of the resistance modules within transposable elements is of a great importance, since these elements contribute highly in dissemination of the resistance determinants among diverse plasmids co-residing in a single bacterial cell.

### Motility

Many bacteria exhibit motility and most of them move by the use of flagella. Motility is defined as the ability to move actively, employing ion motive forces to power flagellar rotation. This is an adaptive trait that is advantageous to bacteria. Motile bacteria have the capacity to move toward favorable niches and avoid detrimental conditions, which allows them to succeed in competition with other groups of microorganisms (Duan et al., 2013; Guttenplan and Kearns, 2013).

Upon analyzing the distribution of genes encoding proteins of different COG categories within the pPSYCH genomes we found that 33 (70.2%) of the proteins classified in the N (cell motility) category are encoded by plasmid pOA238_118 of *O. arcticus* 238. This plasmid carries three clusters of genes that are possibly involved in the formation of flagella. However, the host strain of pOA238_118, as well as other strains of the genus *Octadecabacter*, are non-motile, so the role of the identified gene clusters remains unknown (Vollmers et al., 2013).

A second mechanism enabling “motility” of bacterial cells may be determined by another plasmid (pOA238_160) of *O. arcticus* 238. This replicon carries a *gvp* gene cluster encoding proteins that mediate the formation of gas vesicles (Vollmers et al., 2013). The presence of gas vesicles in polar heterotrophic bacteria may constitute an important selective advantage, since they provide buoyancy which regulates bacterial position in vertically stratified water columns and therefore functions as a dispersal mechanism (Gosink and Staley, 1995).

### Protection against exogenous DNA (restriction-modification and CRISPR-Cas systems)

Bacteria have developed several mechanisms to protect their cells against invading DNA of bacteriophages. The most common are restriction-modification (R-M) and CRISPR-Cas (clustered regularly interspaced short palindromic repeats) systems. In general, the R-M systems are composed of two sequence-specific DNA recognition enzymes—a restriction endonuclease and a methyltransferase. The presence of R-M modules allows horizontally-acquired foreign DNA (non-methylated and so not protected against restriction) to be discriminated from the fully methylated DNA of the host. CRISPR-Cas systems recognize and inactivate exogenous genetic elements by crRNA-guided Cas proteins (Tock and Dryden, 2005; van Der Oost et al., 2014).

**Figure 6:**
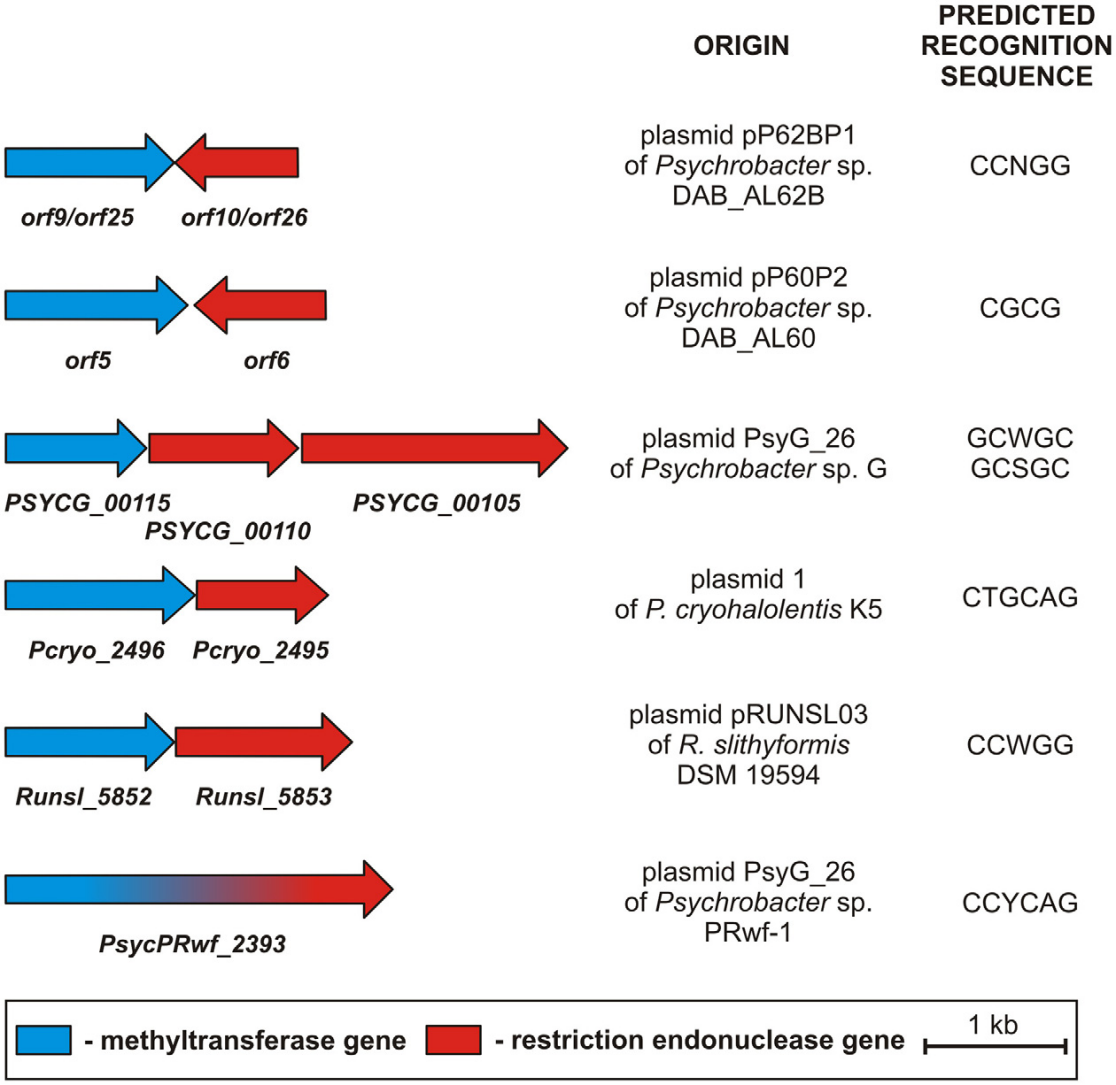
**Genetic organization and the predicted recognition sequences of type II R-M systems carried by pPSYCH plasmids**.

Both systems may play a dual role in bacteria inhabiting extreme environments. Not only do they protect their host strains against infecting bacteriophages, they could also help to eliminate any other invading selfish DNAs (including plasmids), which may consume valuable cellular energy and substrates for their own maintenance. Within the analyzed pPSYCH plasmids we found 7 putative type II R-M modules. The majority of these (6) were present within five plasmids occurring in bacteria of the genus *Psychrobacter*: (i) pP62BP1 (carries two, nearly identical R-M modules), (ii) pP60P2, (iii) plasmid 1, (iv) plasmid PsyG_26 and (v) pRWF101 (Table 1). The remaining type II R-M system was identified within plasmid pRUNSL03 of *R. slithyformis* DSM 19594 (Figure 6).

Six of the identified R-M systems encode DNA m^5^C methyltransferases and have been classified into subtype IIP, while one R-M module (of plasmid pRWF101), encoding only one polypeptide with cleavage and modification domains, belongs to the subtype IIG (Pingoud et al., 2005). The likely recognition sequences of the restriction endonucleases and methyltransferases, predicted on the basis of detailed comparative sequence analyses performed at the REBASE database, are shown in Figure 6.

It is highly probable that the identified R-M systems, besides protecting the cell against foreign DNA, may also function as a type of post-segregational cell killing system that increases the stability of the plasmids in the bacterial population. Such activity has been previously demonstrated for several plasmid-encoded R-M modules (e.g., Dziewit et al., 2011).

**Figure 7:**
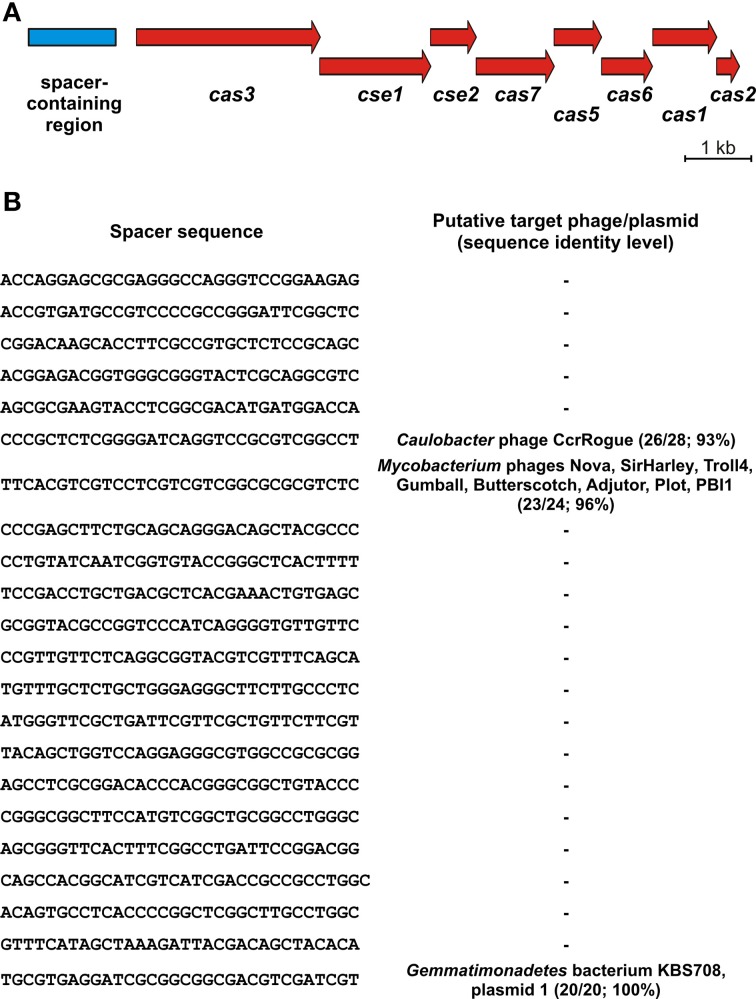
**Genetic organization (A) and nucleotide sequences of the spacers (B) of the CRISPR-Cas *locus* of *Streptomyces* sp. plasmid pSP01**.

The CRISPR-Cas systems mediating immunity to foreign DNA sequences that are integrated as spacers between repeats in the CRISPR *locus*, are widely distributed among bacterial genomes. Our analysis revealed that only one pPSYCH plasmid (pSP01 of *Streptomyces* sp. PAMC26508; Table 1) carries such a system, classified as type I-E (Makarova et al., 2011). The identified *locus* is composed of 8 genes encoding Cas/Cse proteins and the upstream region containing 22 short spacer sequences (21 of 32 bp in length and 1 of 33 bp) (Figure 7). BLASTn comparative analysis revealed that two of the spacers show sequence similarity to genomic sequences of *Caulobacter* and *Mycobacterium* phages and one spacer shows similarity to plasmid 1 of *Gemmatimonadetes* bacterium KBS708 (Figure 7).

## Conclusions

This study represents the first comprehensive survey of plasmids (pPSYCH) maintained by psychrophilic and psychrotolerant bacteria. Our analysis revealed the presence of 66 such replicons occurring in 39 cold-active bacteria isolated from different geographical regions. The pPSYCH plasmids differ greatly in their genetic content. A thorough comparative analysis and the classification of the plasmid-encoded proteins into appropriate COG functional categories has identified several adaptive mechanisms that may benefit bacteria inhabiting cold environments.

We found that the plasmids of cold-active bacteria encode different types of predicted cold shock proteins as well as DNA-repair proteins enabling the cells to cope with enhanced UV radiation. Moreover, the plasmids encode numerous proteins possibly involved in energy production (including hydrogenases), transport of carbohydrates and amino acids, and amino acid synthesis and degradation, which may represent a form of adaptation to nutrient deprivation. They also contain genes conferring resistance to phage infection, heavy metals and metalloids, antibiotics, and (in one case) genes encoding catabolic enzymes involved in naphthalene utilization.

The vast majority of the aforementioned phenotypic traits are encoded within self-transmissible or mobilizable plasmids, which enable their dissemination by HGT. Thus, the pPSYCH plasmids may play an important role in the adaptation of psychrophilic and psychrotolerant bacteria to low temperatures and to rapid environmental changes.

## Author contributions

Lukasz Dziewit conceived, designed and performed the analyses, and wrote the first draft of the manuscript. Dariusz Bartosik contributed to the critical revision of the draft manuscript. Both authors read and approved the final manuscript.

### Conflict of interest statement

The authors declare that the research was conducted in the absence of any commercial or financial relationships that could be construed as a potential conflict of interest.
