# Supplementary figures and images for: Plasmids of psychrophilic and psychrotolerant bacteria and their role in adaptation to cold environments
Source: Front Microbiol. 2014 Nov 6;5:596. doi: 10.3389/fmicb.2014.00596 (PMC4224046; doi:10.3389/fmicb.2014.00596)

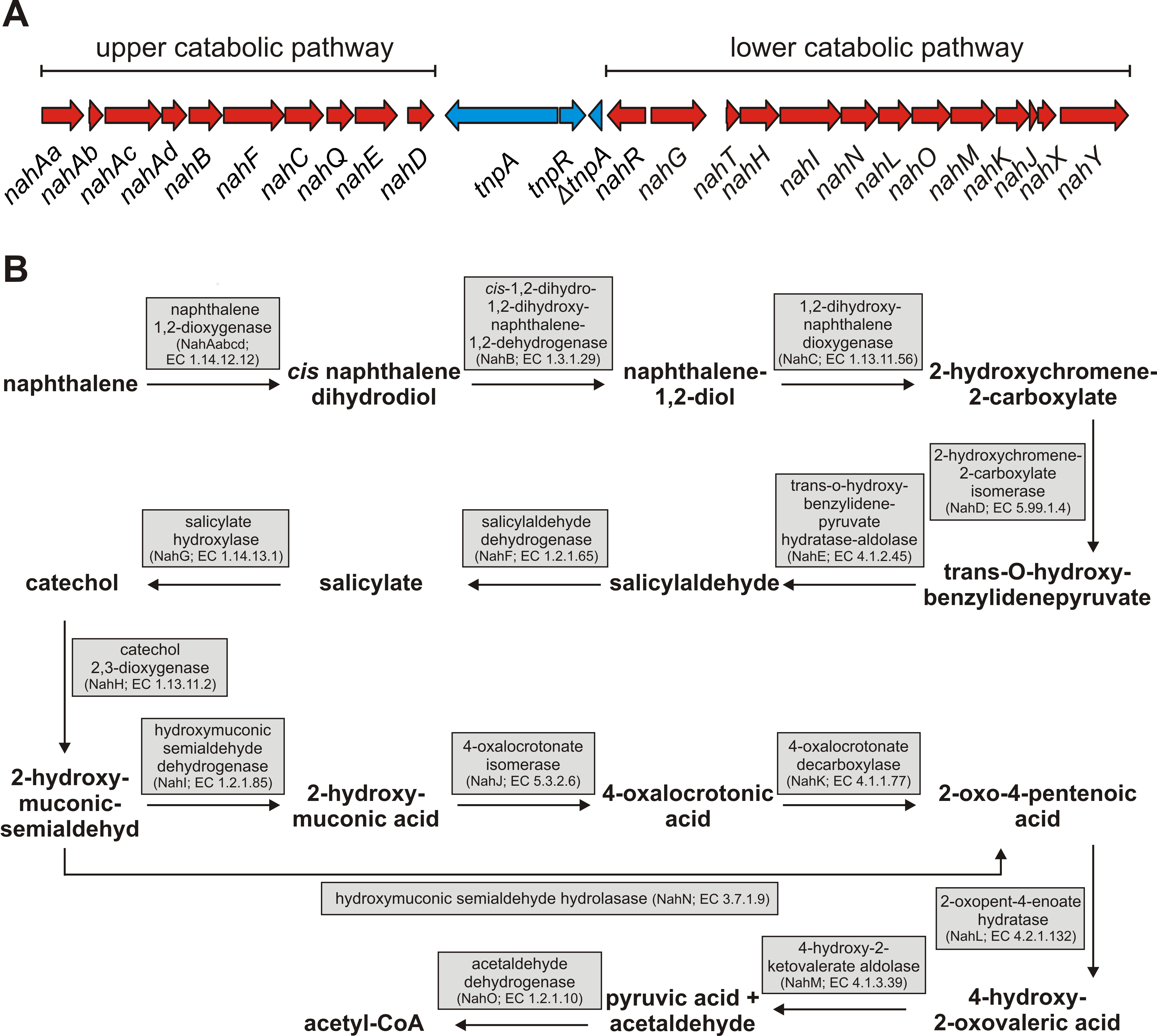

Supplement: Supplementary file 1 [file Image1.TIF]
