# Supplementary material for: Plasmids of psychrophilic and psychrotolerant bacteria and their role in adaptation to cold environments
Source: Front Microbiol. 2014 Nov 6;5:596. doi: 10.3389/fmicb.2014.00596 (PMC4224046; doi:10.3389/fmicb.2014.00596)
Supplement: Supplementary file 2 [file Table1.DOCX]

**Table S1.** Summary of the functional categorization (COG category assignment) of proteins encoded by plasmids of cold-active bacteria.

|  | **pBWB401** | **pBWB402** | **pBWB403** | **pBWB404** | **pCP1** | **pEXIG01** | **pEXIG02** | **pFL1** | **pGIAK1** | **pGLAAG01** | **pGLE121P1** | **pGLE121P2** | **pGLE121P3** | **pKLH80** | **pKW1** | **plasmid 1** | **plasmid F** | **plasmid KOPRI126573** | **plasmid large** | **plasmid PsyG_26** | **plasmid PsyG_3** | **plasmid PsyG_4** |
| --- | --- | --- | --- | --- | --- | --- | --- | --- | --- | --- | --- | --- | --- | --- | --- | --- | --- | --- | --- | --- | --- | --- |
| **[D] Cell cycle control, cell division, chromosome partitioning** | 1 | 1 | 0 | 2 | 0 | 0 | 0 | 0 | 1 | 2 | 0 | 0 | 0 | 0 | 0 | 1 | 0 | 0 | 3 | 0 | 0 | 0 |
| **[M] Cell wall/membrane/envelope biogenesis** | 9 | 8 | 1 | 2 | 0 | 0 | 0 | 0 | 2 | 15 | 0 | 0 | 0 | 1 | 0 | 0 | 0 | 2 | 12 | 0 | 0 | 0 |
| **[N] Cell motility** | 0 | 0 | 0 | 0 | 0 | 0 | 0 | 0 | 0 | 6 | 0 | 0 | 1 | 0 | 0 | 0 | 0 | 2 | 2 | 0 | 0 | 0 |
| **[O] Post-translational modification, protein turnover and chaperones** | 5 | 0 | 0 | 0 | 0 | 0 | 0 | 0 | 4 | 12 | 0 | 0 | 0 | 0 | 0 | 0 | 6 | 0 | 1 | 0 | 0 | 0 |
| **[T] Signal transduction mechanisms** | 17 | 0 | 0 | 1 | 0 | 0 | 0 | 0 | 1 | 19 | 0 | 0 | 1 | 0 | 0 | 0 | 1 | 5 | 0 | 3 | 0 | 0 |
| **[U] Intracellular trafficking, secretion, and vesicular transport** | 0 | 0 | 0 | 0 | 0 | 0 | 0 | 0 | 1 | 11 | 0 | 0 | 8 | 0 | 0 | 0 | 0 | 6 | 12 | 0 | 0 | 0 |
| **[V] Defense mechanisms** | 6 | 0 | 0 | 0 | 0 | 0 | 0 | 0 | 0 | 2 | 0 | 0 | 0 | 2 | 0 | 0 | 0 | 0 | 0 | 1 | 0 | 0 |
| **[W] Extracellular structures** | 0 | 0 | 0 | 0 | 0 | 0 | 0 | 0 | 0 | 0 | 0 | 0 | 0 | 0 | 0 | 0 | 0 | 0 | 0 | 0 | 0 | 0 |
| **[Y] Nuclear structure** | 0 | 0 | 0 | 0 | 0 | 0 | 0 | 0 | 0 | 0 | 0 | 0 | 0 | 0 | 0 | 0 | 0 | 0 | 0 | 0 | 0 | 0 |
| **[Z] Cytoskeleton** | 0 | 0 | 0 | 0 | 0 | 0 | 0 | 0 | 0 | 0 | 0 | 0 | 0 | 0 | 0 | 0 | 0 | 0 | 0 | 0 | 0 | 0 |
| **[A] RNA processing and modification** | 0 | 0 | 0 | 0 | 0 | 0 | 0 | 0 | 0 | 0 | 0 | 0 | 0 | 0 | 0 | 0 | 0 | 0 | 0 | 0 | 0 | 0 |
| **[B] Chromatin structure and dynamics** | 0 | 0 | 0 | 0 | 0 | 0 | 0 | 0 | 0 | 0 | 0 | 0 | 0 | 0 | 0 | 0 | 0 | 0 | 0 | 0 | 0 | 0 |
| **[J] Translation, ribosomal structure and biogenesis** | 2 | 0 | 0 | 0 | 0 | 0 | 0 | 0 | 0 | 1 | 0 | 0 | 0 | 1 | 0 | 1 | 1 | 0 | 0 | 0 | 0 | 0 |
| **[K] Transcription** | 29 | 1 | 0 | 2 | 0 | 0 | 0 | 0 | 6 | 21 | 0 | 2 | 2 | 1 | 0 | 1 | 2 | 6 | 4 | 2 | 0 | 0 |
| **[L] Replication, recombination and repair** | 13 | 5 | 9 | 2 | 1 | 0 | 0 | 0 | 1 | 20 | 0 | 2 | 4 | 7 | 0 | 4 | 1 | 5 | 7 | 6 | 1 | 2 |
| **[C] Energy production and conversion** | 4 | 0 | 1 | 0 | 0 | 0 | 0 | 0 | 1 | 12 | 0 | 0 | 0 | 0 | 0 | 0 | 5 | 8 | 4 | 1 | 0 | 0 |
| **[E] Amino acid transport and metabolism** | 14 | 1 | 1 | 1 | 0 | 0 | 0 | 0 | 0 | 3 | 0 | 0 | 0 | 0 | 0 | 1 | 0 | 2 | 0 | 0 | 0 | 0 |
| **[F] Nucleotide transport and metabolism** | 1 | 1 | 4 | 0 | 0 | 0 | 0 | 0 | 0 | 0 | 0 | 0 | 0 | 0 | 0 | 0 | 0 | 1 | 0 | 0 | 0 | 0 |
| **[G] Carbohydrate transport and metabolism** | 5 | 1 | 0 | 0 | 0 | 0 | 0 | 0 | 0 | 1 | 0 | 0 | 0 | 1 | 0 | 0 | 1 | 0 | 2 | 0 | 0 | 0 |
| **[H] Coenzyme transport and metabolism** | 1 | 0 | 1 | 0 | 0 | 0 | 0 | 0 | 0 | 3 | 0 | 0 | 0 | 0 | 0 | 0 | 0 | 2 | 2 | 1 | 0 | 0 |
| **[I] Lipid transport and metabolism** | 2 | 0 | 0 | 0 | 0 | 0 | 0 | 0 | 0 | 5 | 0 | 0 | 1 | 0 | 0 | 1 | 1 | 4 | 1 | 0 | 0 | 0 |
| **[P] Inorganic ion transport and metabolism** | 3 | 0 | 0 | 0 | 0 | 0 | 0 | 0 | 6 | 21 | 0 | 0 | 0 | 0 | 0 | 2 | 2 | 4 | 0 | 2 | 0 | 0 |
| **[Q] Secondary metabolites biosynthesis, transport and catabolism** | 17 | 0 | 0 | 0 | 0 | 0 | 0 | 0 | 0 | 3 | 0 | 0 | 0 | 0 | 0 | 0 | 1 | 8 | 1 | 0 | 0 | 0 |
| **[R] General function prediction only** | 18 | 1 | 0 | 4 | 0 | 0 | 0 | 0 | 3 | 17 | 1 | 2 | 3 | 0 | 0 | 6 | 3 | 10 | 4 | 0 | 0 | 0 |
| **[S] Function unknown** | 11 | 1 | 3 | 5 | 0 | 1 | 0 | 0 | 0 | 11 | 0 | 1 | 1 | 0 | 0 | 10 | 1 | 1 | 5 | 1 | 0 | 0 |
|  |  |  |  |  |  |  |  |  |  |  |  |  |  |  |  |  |  |  |  |  |  |  |
|  | **plasmid small** | **pMtBL** | **pMWHK1** | **pOA238_118** | **pOA238_160** | **pOA307_63** | **pP109bwP1** | **pP12P1** | **pP32BP1** | **pP43BP1** | **pP43BP2** | **pP43BP3** | **pP43BP4** | **pP60P1** | **pP60P2** | **pP62BP1** | **pPBPR1** | **pPS1M3** | **pRUNSL01** | **pRUNSL02** | **pRUNSL03** | **pRUNSL04** |
| **[D] Cell cycle control, cell division, chromosome partitioning** | 0 | 0 | 0 | 2 | 2 | 1 | 0 | 0 | 0 | 0 | 0 | 0 | 0 | 0 | 0 | 1 | 2 | 0 | 1 | 2 | 1 | 2 |
| **[M] Cell wall/membrane/envelope biogenesis** | 0 | 0 | 0 | 3 | 13 | 2 | 0 | 0 | 0 | 0 | 0 | 0 | 0 | 0 | 0 | 0 | 0 | 0 | 1 | 6 | 1 | 2 |
| **[N] Cell motility** | 0 | 0 | 0 | 33 | 0 | 0 | 0 | 0 | 0 | 0 | 0 | 0 | 0 | 0 | 0 | 1 | 0 | 0 | 0 | 0 | 0 | 0 |
| **[O] Post-translational modification, protein turnover and chaperones** | 1 | 0 | 0 | 3 | 4 | 0 | 0 | 0 | 0 | 0 | 0 | 0 | 0 | 0 | 1 | 0 | 0 | 0 | 5 | 0 | 0 | 2 |
| **[T] Signal transduction mechanisms** | 0 | 0 | 2 | 3 | 0 | 1 | 0 | 0 | 0 | 0 | 0 | 0 | 0 | 0 | 0 | 0 | 0 | 0 | 9 | 2 | 0 | 3 |
| **[U] Intracellular trafficking, secretion, and vesicular transport** | 1 | 0 | 0 | 13 | 0 | 0 | 0 | 0 | 0 | 0 | 0 | 0 | 0 | 0 | 0 | 0 | 2 | 0 | 1 | 4 | 2 | 1 |
| **[V] Defense mechanisms** | 0 | 0 | 0 | 1 | 0 | 1 | 0 | 0 | 0 | 0 | 0 | 0 | 0 | 0 | 0 | 0 | 0 | 0 | 2 | 3 | 1 | 2 |
| **[W] Extracellular structures** | 0 | 0 | 0 | 0 | 0 | 0 | 0 | 0 | 0 | 0 | 0 | 0 | 0 | 0 | 0 | 0 | 0 | 0 | 0 | 0 | 0 | 0 |
| **[Y] Nuclear structure** | 0 | 0 | 0 | 0 | 0 | 0 | 0 | 0 | 0 | 0 | 0 | 0 | 0 | 0 | 0 | 0 | 0 | 0 | 0 | 0 | 0 | 0 |
| **[Z] Cytoskeleton** | 0 | 0 | 0 | 0 | 0 | 0 | 0 | 0 | 0 | 0 | 0 | 0 | 0 | 0 | 0 | 0 | 0 | 0 | 0 | 0 | 0 | 0 |
| **[A] RNA processing and modification** | 0 | 0 | 0 | 0 | 0 | 0 | 0 | 0 | 0 | 0 | 0 | 0 | 0 | 0 | 0 | 0 | 0 | 0 | 0 | 0 | 0 | 0 |
| **[B] Chromatin structure and dynamics** | 0 | 0 | 0 | 0 | 0 | 0 | 0 | 0 | 0 | 0 | 0 | 0 | 0 | 0 | 0 | 0 | 0 | 0 | 0 | 0 | 0 | 0 |
| **[J] Translation, ribosomal structure and biogenesis** | 0 | 0 | 0 | 0 | 0 | 0 | 0 | 0 | 0 | 0 | 0 | 0 | 0 | 0 | 0 | 0 | 0 | 0 | 1 | 0 | 0 | 0 |
| **[K] Transcription** | 0 | 0 | 0 | 5 | 13 | 7 | 0 | 0 | 0 | 0 | 0 | 0 | 0 | 0 | 0 | 3 | 1 | 0 | 6 | 7 | 1 | 2 |
| **[L] Replication, recombination and repair** | 1 | 0 | 1 | 15 | 26 | 3 | 0 | 1 | 0 | 1 | 2 | 2 | 0 | 0 | 3 | 6 | 8 | 0 | 2 | 9 | 5 | 2 |
| **[C] Energy production and conversion** | 0 | 0 | 0 | 0 | 9 | 2 | 0 | 0 | 0 | 0 | 0 | 0 | 0 | 0 | 0 | 2 | 0 | 0 | 7 | 0 | 0 | 2 |
| **[E] Amino acid transport and metabolism** | 0 | 0 | 0 | 0 | 8 | 12 | 0 | 0 | 0 | 0 | 0 | 0 | 0 | 0 | 0 | 1 | 0 | 0 | 1 | 1 | 0 | 0 |
| **[F] Nucleotide transport and metabolism** | 0 | 0 | 0 | 0 | 0 | 0 | 0 | 0 | 0 | 0 | 0 | 0 | 0 | 0 | 0 | 0 | 0 | 0 | 0 | 0 | 1 | 1 |
| **[G] Carbohydrate transport and metabolism** | 0 | 0 | 0 | 0 | 24 | 2 | 0 | 0 | 0 | 0 | 0 | 0 | 0 | 0 | 0 | 0 | 0 | 0 | 13 | 0 | 0 | 0 |
| **[H] Coenzyme transport and metabolism** | 0 | 0 | 0 | 0 | 1 | 2 | 0 | 0 | 0 | 0 | 0 | 0 | 0 | 0 | 0 | 0 | 0 | 0 | 2 | 1 | 1 | 0 |
| **[I] Lipid transport and metabolism** | 0 | 0 | 0 | 2 | 9 | 4 | 0 | 0 | 0 | 0 | 0 | 0 | 0 | 0 | 0 | 1 | 0 | 0 | 4 | 0 | 0 | 0 |
| **[P] Inorganic ion transport and metabolism** | 0 | 0 | 0 | 1 | 1 | 8 | 0 | 0 | 0 | 0 | 0 | 0 | 0 | 0 | 0 | 0 | 0 | 0 | 9 | 9 | 0 | 5 |
| **[Q] Secondary metabolites biosynthesis, transport and catabolism** | 0 | 0 | 0 | 0 | 10 | 4 | 0 | 0 | 0 | 0 | 0 | 0 | 0 | 0 | 0 | 2 | 0 | 0 | 1 | 1 | 0 | 0 |
| **[R] General function prediction only** | 0 | 0 | 0 | 4 | 15 | 7 | 0 | 0 | 1 | 0 | 0 | 0 | 0 | 0 | 2 | 0 | 3 | 0 | 10 | 7 | 6 | 7 |
| **[S] Function unknown** | 0 | 0 | 0 | 5 | 5 | 3 | 0 | 0 | 0 | 0 | 0 | 3 | 0 | 1 | 0 | 1 | 1 | 0 | 2 | 0 | 0 | 1 |
|  |  |  |  |  |  |  |  |  |  |  |  |  |  |  |  |  |  |  |  |  |  |  |
|  | **pRUNSL05** | **pRWF101** | **pRWF102** | **pSCD** | **pSFKW33** | **pSinA** | **pSM327** | **pSM429** | **pSP01** | **pTA144 Dw** | **pTA144 Up** | **pVSAL111** | **pVSAL320** | **pVSAL43** | **pVSAL54** | **pVSAL68** | **pVSAL840** | **pWNCR12** | **pWNCR15** | **pWNCR47** | **pWNCR64** | **pWNCR9** |
| **[D] Cell cycle control, cell division, chromosome partitioning** | 1 | 0 | 0 | 1 | 0 | 1 | 0 | 0 | 1 | 0 | 0 | 0 | 1 | 0 | 0 | 0 | 1 | 1 | 0 | 1 | 2 | 0 |
| **[M] Cell wall/membrane/envelope biogenesis** | 3 | 0 | 0 | 5 | 0 | 0 | 0 | 0 | 4 | 0 | 0 | 0 | 0 | 0 | 0 | 0 | 2 | 2 | 1 | 1 | 3 | 0 |
| **[N] Cell motility** | 0 | 0 | 0 | 0 | 0 | 2 | 0 | 0 | 0 | 0 | 0 | 0 | 0 | 0 | 0 | 0 | 0 | 0 | 0 | 0 | 0 | 0 |
| **[O] Post-translational modification, protein turnover and chaperones** | 0 | 0 | 0 | 4 | 0 | 1 | 0 | 0 | 0 | 0 | 0 | 0 | 0 | 0 | 0 | 0 | 0 | 0 | 0 | 0 | 0 | 1 |
| **[T] Signal transduction mechanisms** | 1 | 0 | 0 | 6 | 0 | 10 | 0 | 0 | 1 | 0 | 0 | 0 | 0 | 0 | 0 | 0 | 0 | 0 | 0 | 1 | 4 | 0 |
| **[U] Intracellular trafficking, secretion, and vesicular transport** | 0 | 0 | 0 | 2 | 0 | 12 | 0 | 0 | 2 | 0 | 0 | 0 | 0 | 0 | 0 | 0 | 2 | 2 | 0 | 1 | 2 | 0 |
| **[V] Defense mechanisms** | 0 | 1 | 0 | 3 | 0 | 0 | 0 | 0 | 3 | 0 | 0 | 0 | 0 | 0 | 0 | 0 | 0 | 0 | 0 | 1 | 0 | 0 |
| **[W] Extracellular structures** | 0 | 0 | 0 | 0 | 0 | 0 | 0 | 0 | 0 | 0 | 0 | 0 | 0 | 0 | 0 | 0 | 0 | 0 | 0 | 0 | 0 | 0 |
| **[Y] Nuclear structure** | 0 | 0 | 0 | 0 | 0 | 0 | 0 | 0 | 0 | 0 | 0 | 0 | 0 | 0 | 0 | 0 | 0 | 0 | 0 | 0 | 0 | 0 |
| **[Z] Cytoskeleton** | 0 | 0 | 0 | 0 | 0 | 0 | 0 | 0 | 0 | 0 | 0 | 0 | 0 | 0 | 0 | 0 | 0 | 0 | 0 | 0 | 0 | 0 |
| **[A] RNA processing and modification** | 0 | 0 | 0 | 0 | 0 | 0 | 0 | 0 | 0 | 0 | 0 | 0 | 0 | 0 | 0 | 0 | 0 | 0 | 0 | 0 | 0 | 0 |
| **[B] Chromatin structure and dynamics** | 0 | 0 | 0 | 0 | 0 | 0 | 0 | 0 | 0 | 0 | 0 | 0 | 0 | 0 | 0 | 0 | 0 | 0 | 0 | 0 | 0 | 0 |
| **[J] Translation, ribosomal structure and biogenesis** | 0 | 0 | 0 | 0 | 0 | 1 | 0 | 0 | 1 | 0 | 0 | 0 | 0 | 0 | 0 | 0 | 0 | 0 | 0 | 0 | 0 | 0 |
| **[K] Transcription** | 1 | 0 | 0 | 6 | 1 | 15 | 0 | 0 | 1 | 0 | 0 | 0 | 2 | 0 | 0 | 0 | 1 | 0 | 2 | 3 | 5 | 0 |
| **[L] Replication, recombination and repair** | 3 | 1 | 1 | 4 | 1 | 8 | 1 | 0 | 4 | 0 | 0 | 1 | 3 | 1 | 0 | 0 | 9 | 5 | 2 | 8 | 6 | 1 |
| **[C] Energy production and conversion** | 2 | 0 | 0 | 16 | 0 | 4 | 0 | 0 | 1 | 0 | 0 | 0 | 0 | 0 | 0 | 0 | 0 | 0 | 0 | 0 | 0 | 0 |
| **[E] Amino acid transport and metabolism** | 2 | 0 | 0 | 1 | 0 | 1 | 0 | 0 | 1 | 0 | 0 | 0 | 0 | 0 | 0 | 0 | 1 | 0 | 0 | 1 | 1 | 0 |
| **[F] Nucleotide transport and metabolism** | 0 | 0 | 0 | 0 | 0 | 0 | 0 | 0 | 2 | 0 | 0 | 0 | 0 | 0 | 0 | 0 | 0 | 0 | 0 | 1 | 1 | 1 |
| **[G] Carbohydrate transport and metabolism** | 5 | 0 | 0 | 1 | 0 | 1 | 0 | 0 | 0 | 0 | 0 | 0 | 0 | 0 | 0 | 0 | 1 | 0 | 0 | 0 | 5 | 0 |
| **[H] Coenzyme transport and metabolism** | 0 | 0 | 0 | 5 | 0 | 1 | 0 | 0 | 0 | 0 | 0 | 0 | 1 | 0 | 0 | 0 | 0 | 0 | 0 | 0 | 0 | 0 |
| **[I] Lipid transport and metabolism** | 1 | 0 | 0 | 1 | 0 | 0 | 0 | 0 | 0 | 0 | 0 | 1 | 0 | 1 | 1 | 0 | 1 | 0 | 0 | 0 | 0 | 0 |
| **[P] Inorganic ion transport and metabolism** | 0 | 0 | 0 | 10 | 0 | 22 | 0 | 0 | 1 | 0 | 0 | 0 | 3 | 0 | 0 | 0 | 0 | 1 | 5 | 0 | 10 | 0 |
| **[Q] Secondary metabolites biosynthesis, transport and catabolism** | 1 | 0 | 0 | 0 | 0 | 1 | 0 | 0 | 0 | 0 | 0 | 0 | 0 | 0 | 0 | 0 | 1 | 0 | 0 | 0 | 1 | 0 |
| **[R] General function prediction only** | 9 | 1 | 0 | 6 | 0 | 3 | 0 | 0 | 6 | 0 | 0 | 0 | 0 | 0 | 0 | 0 | 3 | 0 | 1 | 1 | 4 | 0 |
| **[S] Function unknown** | 4 | 1 | 0 | 3 | 0 | 4 | 0 | 0 | 2 | 0 | 0 | 0 | 1 | 0 | 0 | 0 | 3 | 1 | 1 | 3 | 5 | 0 |
